# Close ties: an exploratory Colored Eco-Genetic Relationship Map (CEGRM) study of social connections of men in Familial Testicular Cancer (FTC) families

**DOI:** 10.1186/1897-4287-10-2

**Published:** 2012-03-01

**Authors:** June A Peters, Regina Kenen, Lindsey M Hoskins, Gladys M Glenn, Christian Kratz, Mark H Greene

**Affiliations:** 1 Clinical Genetics Branch, Division of Cancer Epidemiology and Genetics, National Cancer Institute, National Institutes of Health, US Department of Health and Human Services, 6120 Executive Blvd, Rockville, MD, 20852 USA; 2 Department of Sociology and Anthropology, The College of New Jersey, Ewing, NJ, USA

## Abstract

**Background:**

Testicular cancer, while rare compared with other adult solid tumors, is the most common cancer in young men in northern Europe and North America. Risk factors include white race, positive family history, contralateral testicular cancer, cryptorchidism, infertility and testicular microlithiasis. As the genetic causes of familial clusters (Familial Testicular Cancer or FTC) are being sought, it is also important to understand the psycho-social experiences of members of FTC families.

**Methods:**

This is a cross-sectional examination via the Colored Eco-Genetic Relationship Map (CEGRM) of social connections reported by 49 men in FTC families participating in NCI research study 02-C-178.

**Results:**

The CEGRM was acceptable and feasible for use with men in FTC families, and valuable in understanding their social connections. These men have largely adjusted to the TC history in themselves and/or their relatives. They have considerable social and emotional support from family and friends, although there is wide variability in sources and types.

**Conclusions:**

The CEGRM focuses on men's social connections and close emotional bonds in FTC families. This action-oriented process of placing colored symbols on significant relationships uncovered previously under-appreciated emotions accompanying men's social exchanges. Most men in FTC families succeed in re-establishing a sense of normalcy in their lives and social connections, in the aftermath of a testicular cancer diagnosis.

## Introduction

Testicular Cancer (TC) is rare, with about 8,500 new US cases per year in 2010 according to the American Cancer Society website (accessed 11/28/2011) (http://www.cancer.org/acs/groups/content/@epidemiologysurveilance/documents/document/acspc-026238.pdf). Worldwide, 2008 GloboCan statistics estimated 52,322 TC cases worldwide http://globocan.iarc.fr/factsheets/populations/factsheet.asp?uno=900#MEN (accessed 11-21-11), which is far less than incidence of common cancers such as lung, which is projected to develop more than one million new cases among men, worldwide.

Unlike the common cancers of aging, testicular cancer is most common in young men (typically, aged 15-35 years) in northern and western Europe and North America. Of concern, TC incidence has been increasing since World War II [1]. Testicular cancer is highly treatable, but chemotherapy treatment may leave long-lasting adverse effects. Known TC risk factors include white race, prior contralateral testicular cancer, cryptorchidism and other genitourinary (GU) abnormalities, sub-fertility and family history. Other suspected factors include tall adult height, early exposures to female hormones and/or endocrine disruptors and, possibly, testicular microlithiasis [2,3].

Testicular cancer (TC) is usually not the first condition that comes to mind when considering hereditary contributions to cancer susceptibility. In addition to TC being a rare cancer, only a small percentage, i.e., about 2% of men with TC have an affected relative and are considered to have Familial Testicular Cancer (FTC). Having a positive family history of TC is associated with a 4-6 fold increase in sons of TC-affected fathers; and 8-10 fold increase in brothers [4,5]. These relative risks are higher than other known hereditary cancer susceptibility syndromes, in which a positive family history confers an approximately 2-fold increase.

Unfortunately, there is currently no clinical genetic testing available for FTC, unlike other hereditary cancer susceptibility syndromes in which highly-penetrant gene mutations have been discovered as the genetic cause. More detailed information about our current understanding of FTC genetic and environmental risk factors of FTC can be found in Additional file 1 and elsewhere.

While the specific genetic causes of familial testicular cancer clusters (FTC) are being sought, it is equally important to understand the experiences and quality-of-life of FTC family members, to better help the family cope with TC or reduce the risk of future cancer. In a recent literature search, we found no studies focusing on familial factors related to psychosocial or behavioral adjustment to TC, although the literature has described effects of sporadic TC on sexual functioning and marital relationships [6,7]. While some men with TC and their spouses have reported problematic couples' adjustment in the first year after treatment, long-term adjustment of couples was collectively positive in terms of good communication, spousal support, and marital satisfaction; only a minority of couples continued to have relationship difficulties. There are inconsistencies in international studies of Japanese and French TC patients in terms of anxiety, depression, relationships and economic consequences of having TC [8,9]. We were unable to find any publications evaluating the social consequences of TC, beyond the couple level.

One focus of the current study is to learn more about men who become involved in cancer genetics studies. Most of the psychosocial literature about gender differences in reactions to hereditary cancers comes from families with Hereditary Breast Ovarian Cancer (HBOC) susceptibility and Lynch syndrome (formerly HNPCC). The male participants in the HBOC studies were primarily mutation carriers with only modestly increased cancer risk, non-carrier male relatives, or spouses. For example, we know that men in HBOC families worry about their sisters, partners, or children [10-14]. In another study, predictors of distress in male *BRCA1/2 *carriers at 1 year included higher baseline cancer-specific distress and being unmarried [15]. A number of studies have found that men in HBOC families report communication difficulties [16-19]. In prior HBOC studies from our group, we observed complex health communication processes between male and female relatives in HBOC families, with more men identified as "blocking" health communications, but both men and women contributing to health communication impediments [20,21].

Because of the dearth of data about social functioning in men with FTC, we examined supportive social exchanges in men using methods comparable to those with which we evaluated the social networks of women in HBOC families. To capture relevant social exchanges between our study participants and their relatives, friends, and significant non-kin, we included the Colored Eco-Genetic Relationship Map (CEGRM) in the FTC study. This is a simple method of obtaining detailed social exchange information through an interactive process. During the conjoint construction of a CEGRM, the participants and investigators explore 4 social interaction domains of information, tangible, emotional, and spiritual/religious support. The result of this process is a concise, visual representation of these social exchange domains with color-coded symbols applied to the genetic pedigree [22]. Our publications over the past decade have demonstrated that the CEGRM is feasible, comfortable and informative tool when used with women in the clinical cancer genetics research context to facilitate better understanding of the social and emotional sequelae of belonging to an HBOC family [19,23-25]. Driessnack utilized a modified CEGRM process with similarly positive results in a pilot study of children aimed at understanding their sources of, and perceptions about, health information [26].

**Figure 1:**
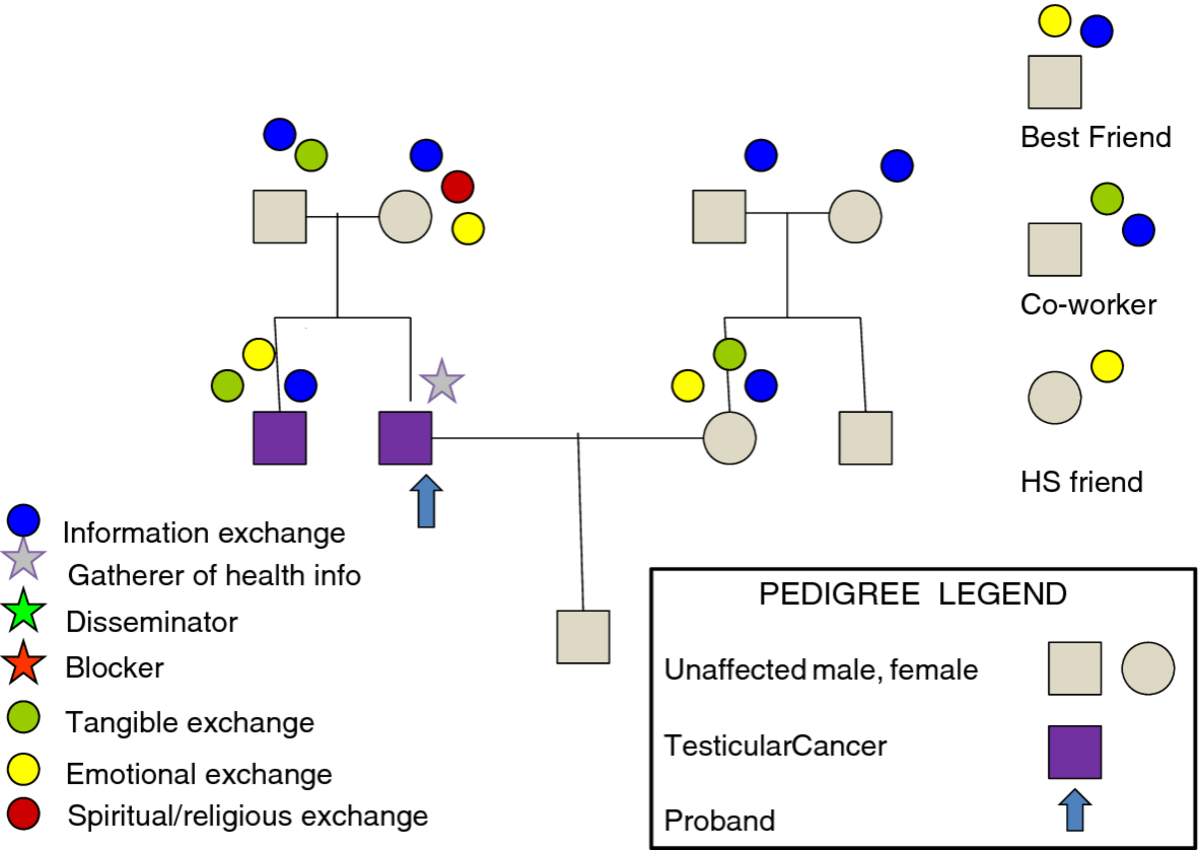
**Sample CEGRM**.

Figure 1 illustrates a sample CEGRM. One goal in this study was to extend the CEGRM to men, to determine if this tool was informative among male interviewees. We hypothesized that men would be less interested in, or perhaps even resistant to, the effort required to complete the CEGRM.

### Aims

We report here our efforts to:

1. Describe characteristics of men participating in FTC CEGRM study of social networks;

2. Determine acceptability, feasibility and utility of CEGRM assessment in a clinical research population of men;

3. Explore other themes relevant to social and gender-role functioning of men and women within FTC families.

## Methods

### Study design

This is an exploratory, cross-sectional social assessment of men in FTC families at the time of their clinical visits on our NIH research protocol 02-C-0178 between 2003 and 2011. This is not designed to be a family systems study, but rather a social assessment within a familial cancer research context. Both descriptive quantitative feasibility data and qualitative data analyses are included.

### Participants and recruitment

The current study was conducted among members of families enrolled in an IRB-approved, NCI-sponsored multidisciplinary etiologic study of Familial Testicular Cancer (NCI Protocol 02-C-0178; NCT-00039598). Families were eligible if they had 2 or more confirmed cases of TC or a single bilateral case. Men with history of TC, their first-degree relatives and spouses were invited to clinic for intensive medical evaluations. A detailed description of the parent study is available elsewhere [3]. After giving written informed consent, all participants completed mailed questionnaires about relevant personal and family characteristics. We invited a subset to the NIH Clinical Center for clinical and psychosocial evaluation.

### Study population

The current study of 49 consecutive male participants (5 female relatives or spouses were seen but excluded from analysis) was conducted in the NIH Clinical Center from 2005 to April 2011. Early in the study, we were seeing both male and female family members; however, this practice was dropped in 2008, when clinical evaluation in the parent protocol was re-focused on male participants with history of microlithiasis.

### Procedures

#### Data collection

Prior to the NIH visit, each FTC study participant completed Lifestyle and Attitude Questionnaires (LAQ), which included demographics and a number of standardized scales as well as several study-specific psychosocial/behavioral measures. The LAQs were customized for men with a history of TC (LAQMH), relatives at risk (LAQMR), and female relatives and spouses (LAQF).

*Demographic Data *included age, partnered status, race/ethnicity, education, previous TC diagnosis, time since TC diagnosis, relationship to the proband, whether the participant had children and, if so, how many.

#### Psychological distress as measured by the brief symptom inventory-18 (BSI-18)

The BSI-18 is a standardized, frequently-used, highly-sensitive, validated self-report symptom inventory designed to screen for psychological distress [27]. It yields a Global Distress score as well as sub-scores for Somatization, Anxiety and Depression, with reliability measurements between 0.74-0.84 for all scales. Normative T-scores derived from general population norms were computed for each BSI-18 sub-scale.

#### Social assessment via the CEGRM

After written and verbal consent was obtained, study participants who had completed written medical history questionnaires and the LAQs were seen for a clinical visit that included in-person psychological and social assessment, as well as medical history, physical examination and testicular ultrasounds. Each participant conjointly constructed the CEGRM with a female researcher (JP or LH), using the participant's computerized genetic pedigree as a template, as previously described and briefly described below [25].

The researcher used a semi-structured script to guide the participant towards actively identifying, with differently colored symbols, the individuals who filled various social roles in their lives from within and outside the biological family-informational (blue dots), tangible (green dots), emotional (yellow dots), and spiritual/religious (red dots). We were able to count these socially supportive exchanges within and outside the family. Star symbols were used to identify relatives exemplifying the specialized family health communication roles of "information gatherers" (green star), "disseminators" (silver star), and "blockers" (red star); however, these roles were not explored more deeply in this report. The colors corresponding to the different social exchange domains were chosen based on sticker availability.

### Evaluation of the CEGRM process

We evaluated Compliance with research request, Feasibility and Utility of CEGRMs.

### Compliance

Compliance was based on the frequency of accepting vs. rejecting an invitation to complete a CEGRM.

### Quantitative CEGRM feasibility measures

We used the same feasibility measures in this FTC study as we had used in our prior studies with women in the HBOC families [24,25]:

• Time to complete a CEGRM beginning with a previously-constructed genetic pedigree

• Understanding of the CEGRM concept and process*

• Comfort doing the CEGRM*

• Ease of use of the colored stickers*

• Ease of talking about social relationships in the CEGRM context,* and

• Usefulness in eliciting narrative stories*

*self-reported responses of the participants on 1-10 scales with 1 being the most favorable answer and 10 being the least favorable

Finally, participants subjectively estimated:

• The percent of placement of the colored stickers without help

• The proportion of time that they perceived that they talked vs. the time that the investigator talked

From the CEGRM data we were also able to tabulate types of socially supportive exchanges (information, tangible, emotional, spiritual) and sources of support, both within and outside biological families.

### Utility: collection, coding and analysis of qualitative data

To achieve a more in-depth understanding of the participants' experiences and social connections, we collected qualitative data from CEGRM notes. While the participant was constructing the CEGRM by placing the colored symbols, the investigator took detailed handwritten notes (including salient points from participants' verbal comments, with verbatim quotes whenever possible) to capture the complexity, reasoning, and fine-grained characterization of individual perceptions regarding how and why they were connected socially to various individuals within and beyond the biological family. We used an iterative process of identifying and naming recurrent patterns, relationships and processes in the data [28].

The qualitative aspect of this study using data collected during the CEGRM interaction was based on inductive reasoning and an interpretive paradigm. All of the researchers' handwritten notes that were taken during the CEGRM construction and related to the perceived blockers were transcribed. Two researchers (RK & JP) reviewed all CEGRMs and the accompanying notes for the selected participants, using open coding to identify themes. The data related to the breadth of emotionally connected relationships consisted of the yellow dots on the individual to whom the participant designated as having signifying emotional bonds. Investigator handwritten notes regarding participants' descriptive comments about the nature and quality of emotional connections represented the depth of the relationships. These we included in the audit trail that we developed to defend the trustworthiness and creditability of the analysis [29].

## Results

### Study population

Table 1 summarizes the demographic and familial characteristics of our study population. Forty-nine men from 19 families participated in this study. Five female members of these families were seen for CEGRMs but were excluded from this analysis. In general, the female participants were similar demographically to the males, since they were members of the same families.

**Table 1:** Demographics and Familial Characteristics

	*Number*	*Percent*
***Education***	47	96%
***High School ***	6	12%
***College/Grad ***	24	49%
***Prof/Post Grad ***	17	35%
***Marital Status***	49	100%
***Married ***	32	65%
***Single ***	13	27%
***Divorced ***	4	8%
***Relatedness***	49	100%
***Proband ***	15	31%
***FDR ***	32	65%
***SDR ***	1	2%
***TDR ***	1	2%
***Children***	49	100%
***Yes ***	32	65%
***No ***	17	35%
***Avg # ***	1.8 (0-7)	
***Affected TC***	30	61%
***Yes***		
***No ***	19	39%

The average age of male participants was 40 years (range 16-79 years). They were well educated, with 85% having completed at least some college or technical school. The majority of adult men (65%) were married or partnered, 27% were single, and 8% divorced; 63% had children, ranging from 0-7 per participant, with a mean of 2 children per participant.

The majority of families contained 2 affected men with TC, which is by far the most common presentation of FTC [30], although there were a few families with > 2 affected men.

There was also one family with a proband with TC and his sister with germ-cell ovarian cancer [31]. Thirty (61%) participating men had a personal history of TC, half of whom were the family proband. The mean time since TC diagnosis was 10 years, median was 8 years, and mode was 3 years, with a range from 1-30 years from TC diagnosis to study participation. In terms of relatedness, we saw 32 first-degree relatives (FDRs), i.e., mainly brothers, 1 second degree relative (SDR), and 1 third-degree relative (TDR).

### Compliance and feasibility of CEGRM by Men in FTC families

The CEGRM process was very well accepted by the men in this study. Of participants who were invited to participate, 100% accepted. There were also many appreciative comments.

The CEGRMs were facilitated by one of two female investigators (JP or LH). The average length of time required to complete a CEGRM was about 40 minutes (range: 15-70 minutes), reflecting that while some men were reticent, the majority were fully cooperative in the interchange (mode = 30 min.), and a few were verbally expansive.

Table 2 reflects the results that were self-reported by the participants immediately following completion of the CEGRM, by circling a response from 1-10, with 1 being the most favorable score and 10 being the least favorable. All scores were in the strongly-positive range (1.4 to 2.2). The participants placed the stickers themselves 96% of the time; however, there were two participants who were unable to manipulate the stickers due to platinum chemotherapy-related peripheral neuropathy; these two ranked ease of use of stickers as > 5. For this sub-group with poor manual dexterity, colored pencils were the preferred study media. Most participants estimated (the interviews were not timed) that they talked, on average, more than 50% of the time (range: 15-100%).

**Table 2:** CEGRM Assessment Feasibility Measures

*Sex: Male Participants *	*# of Partic*	*Mean*	*Median*	*Mode*	*Min*	*Max*	*Std. Deviation*
***Time to complete (in min.) ***	44	41 min	40 min	30 min	15 min	70 min	14.67
Understanding*	46	1.65	1	1	1	5	0.97
Comfort*	48	1.79	1	1	1	5	1.07
Ease of stickers use*	47	3.04	1	1	1	10	2.55
***Put stickers without help ***	47	95.77%	100%	100%	5%	100%	16.18
Ease of talking*	48	2.08	1	1	1	6	1.46
Encouraged stories*	48	2.00	1	1	1	7	1.50
***Proportion participant talked ***	48	61.23%	60%	50%	15%	100%	17.72

* The range of values was 1-10, with 1 being the most favorable answer, and 10 the least

### Utility of the CEGRM

Table 3 summarizes the participants' different types of social exchanges and health communication roles. The CEGRM process was useful in understanding men's experiences with multiple cases of TC in the family. The men from multiple-case families reported a significant variety of social exchange types. They designated a mean of 7 people (range 1- 19) as exchanging tangible support, 6 (range 1-13) exchanging emotional support and 3-4 (range 0-20) exchanging spiritual/religious support. The spiritual contacts were not evenly distributed; three-quarters of participants designated 0-3 religious connections, whereas 5 men reported a very high level of supportive contacts (e.g., > 12) within their religious communities. The latter participants belonged to strongly traditional faiths e.g., Mormon, Eastern Orthodox or Roman Catholic.

**Table 3:** Types of Social Exchanges

*Type of Social Exchange*	*# of Participants*	*Mean*	*Median*	*Mode*	*Min*	*Max*	*Std. Deviation*
***Health Information***	48	10.12	9	9	2	24	3.47
***Gatherers***	48	1.79	2	1	0	5	2.93
***Disseminators***	48	1.56	1	1	0	4	1.58
***Blockers***	48	0.45	0	0	0	3	1.80
***Tangible help***	48	6.62	5	4	1	19	1.62
***Emotional support***	48	5.88	5	3	1	13	1.34
***Spiritual connection***	48	3.46	2	1	0	20	.68

#### Health communication

Health communication about cancer seemed open and straight-forward, with the exception of several men who self-described themselves as very private people. The mean number of people designated with blue circles for information exchanges was 10, with a wide range (2- 24), even between men from the same families. There were a mean of 1-2 information gatherers and disseminators within most families (gatherers mean = 1.8; disseminators mean = 1.6). In contrast, there was less than one information blocker per male respondent (mean = 0.5). Most of the men seemed generally open about discussing TC within a tight circle of family and/or friends.

In the minority, one retired man reported that he and his wife "like our time alone" and have no friends except old neighbors from a prior home in another state, whom they see once a year. He told us this to explain why he placed social interaction symbols only on himself, his wife, son, and daughter; listing no friends. Another man with few colored symbols saw himself as information-seeking, task-oriented and self-sufficient, with minimal need for friends. One participant spoke of a conscious communication strategy based on compartmentalization, i.e., having defined topics that were appropriate only with specific people, such as talking to one brother about outdoor activities, a friend about sports and a third person about travel. These examples were exceptions rather than the rule.

#### Social support types

Participants had varied social exchanges in health information, tangible, emotional and spiritual domains. They reported most exchanges in health information (mean = 10), followed by tangible and emotional supports. Spiritual exchanges were rare for most men, although this domain ranked high for a small minority. Many men reported friendships with other men, often engaged in activities of mutual interest. A number of men explicitly stated that they found support from groups rather than individuals, e.g., playing on sports teams, interacting with medical care teams, friends, co-workers and neighbors collectively. Our subjects seemed to have come to terms with having had TC; most cancer survivors in our study were many years beyond their diagnosis and treatment (mean = 10 years, range = 1-30 years) and they did not seem to express intense or challenging emotional issues.

As with our other studies in which situational factors affected social connections, we found that some men were more concerned about and actively engaged with other current family health problems, e.g., a sister with learning problems or a wife with cancer, than about their own cancer history [20].

In families with military background or current active duty, we noticed several distinctive characteristics of social engagement: a tendency to trust mainly people within the same military and medical-military culture; a communication style of short, direct statements; and the use of traditional male coping mechanisms of silence, sports, or going for a drink with male buddies.

#### Close ties: emotional ties via CEGRM

We focused on the persons designated as sources of emotional support as one metric of participants' close ties, in part because much of the existing medical psychology literature describes men as lacking emotional discourse regarding health matters with their family and friends. The male participants from FTC families who chose to participate in our study had substantial emotional ties with others both inside and outside their families. In total, the men in our study reported an average of 6 emotionally close individuals within their CEGRM. Of these, about 3-4 designated emotional supports were family members (range = 0-13) versus 2*-*3 outside the family (range 0-8) for a ratio of 2:1 of family to friends. There were slightly more emotionally supportive connections with spouses/female members of the family (average 2.1 female vs. 1.6 male). Table 4 summarizes the participants' sources of emotional supports within and outside the family.

**Table 4:** Emotional Support Sources: Total, Family, Friends, Other

	*# of Participants*	*Mean*	*Median*	*Mode*	*Min*	*Max.*	*Std. Deviation*
***Total Social Support Family:***	42	5.57	4.5	3	1	14	3.47
***Total Familial***							
***Emotional supports***	42	3.62	3	1	0	13	2.93
***Male Relatives***	42	1.57	1	0	0	5	1.58
***Female Relatives***	42	2.10	2.1	1	0	9	1.75
***Friends:***							
***Total Non-Family***							
***Emotional supports***	42	1.90	2	2	0	8	1.62
***Male Friends***	41	1.17	1	1	0	7	1.34
***Female Friends***	41	0.51	0	0	0	3	0.67
***Other:***	41	0.17	0	0	0	2	0.44

Among friends, our participants reported an average of twice as many male as female friends, and a few examples of supports from other sources (e.g., team, running group, basketball buddies, medical team, and pets). The men participating in this study generally related to women in their nurturing roles as wives, mothers, sisters-in-law, and friends, while relating to their brothers, old school friends, close work companions, neighbors or sports team members as confidantes and activity buddies. In contrast, it was rare for our subjects to report close emotional support from fathers, sisters, daughters, sons, and professional contacts.

#### Emotional status on BSI-18 questionnaire

Male FTC family members self-reported minimal emotional distress on the BSI-18, with mean sub-scale and global T scores of approximately 48-49, close to the standardized population mean of 50 (Table 5). The wide range of values for the BSI-18 scores indicates high variability in emotional status. As with many psychosocial studies in genetic settings, while the majority of participants were not clinically distressed, there was a sub-set of individuals with very low or very high distress levels.

**Table 5:** Psychosocial Distress via BSI-18 T-Scores

	N	Mean	Median	Mode	Minimum	Maximum	Std. Deviation
***BSI_SOM_T***	42	47.74	48	40	37	75	8.26
***BSI_DEP_T***	42	49.14	48	42	42	77	9.16
***BSI_ANX_T***	42	49.45	48	39	39	75	8.79
***BSI_GSI_T***	42	49.14	48	45	34	76	8.84

*Raw BSI scores have been converted to standardized T scores, with a mean of 50 and standard deviation of 10

Table 5 provides detailed descriptive BSI-18 data. Male FTC family members self-reported minimal emotional distress on the BSI-18, with mean sub-scale and global T scores of approximately 48-49, close to the standardized population mean of 50. The wide range of values for the BSI-18 scores indicates high variability in emotional status. As with many psychosocial studies in genetic settings, while the majority of participants were not clinically distressed, there was a sub-set of individuals with very low or very high distress levels.

Specifically, there were 6 (12%) men reporting no symptoms in any domain, i.e., T-scores < 40.

On the other end of the spectrum, there were 2 men (4%) reporting very high scores of above 70 (+ 2 SDs) on Global Symptom score (T-scores of 72, 76). Both of these men had personal history of TC and had high scores across all 3 sub-scale measures. There were several other men who had high (T score > 60 = + 1 SD) sub-scale scores but with Global scores less than 60, specifically, 6 of these additional men had high Depression scores, 2 also with high Anxiety scores and 1 additional man reporting high Somatization score in addition to high Depression.

#### Correlation of emotional supports with distress

We found a high positive correlation of 0.5 (*p *= 0.002) between global distress and number of emotional supports. The two men with very high Global BSI scores had 13 and 10 emotional supports apiece. This positive correlation held true for each of the Somatization, Depression and Anxiety subscales as well. There was no significant correlation between BSI-global distress and informational, tangible or spiritual social exchanges.

#### Distress, emotional exchanges and affected status

We compared mean GSI scores (global distress) of men who had a history of prior testicular cancer (Affected) with men who did not (Unaffected). We found that previously affected men had a slightly higher mean global distress score than those without prior testicular cancer (50.79 vs. 45.46); however, this difference was not statistically significant in this small sample of 42 men with BSI scores (*p *= 0.07). The previously affected men also reported slightly more emotional exchanges (6.08 vs. 4.75) than did the unaffected men; however, this difference was not significant (*p *= 0.18).

### Other prominent themes

#### Identity

Most people with cancer have a strong desire for rapid return to a sense of normalcy in their lives. (http://www.cancer.gov/ncicancerbulletin/072611/page6). For most affected men in our study, normal physical, relational and emotional functioning returned, for others, not. A few of our participants spoke of what we consider a "spoiled" identity, one characterized by social stigma due to cancer [32]. One man said that his father doesn't talk about any 'weaknesses' like cancer saying, "If there is a broken chain in the family, e.g., we sons developed cancer, Dad takes it personally as a failure." In contrast to his father, this man, however, sees himself as a helper and spiritual healer.

Some of the men projected the traditional masculine attitude of being strong and self reliant, but we could not distinguish whether this style predated their TC diagnosis or whether it was a *post hoc *defensive coping mechanism. For a few men, the cancer and/or awareness of aging seem to have opened the door to exploration of existential issues. One man described himself as "like Superman," doing everything for others, but secretly wishing that he had had more support for himself during chemotherapy. He reported that he felt a tension between being "the strong one" and the "family teddy bear". Another man described himself as "very strong forever" until he "fell apart" after dealing with multiple serious health problems; he mentioned that he is still (after more than a year) adjusting emotionally to not being as physically strong as he used to be.

Another participant seemed to reject the cancer survivor identity very consciously, saying, "I'm not a cancer person." Later he stated, "Going through cancer wasn't such a big deal." For many men who had only orchiectomy surgery as treatment, the process from diagnosis to treatment was indeed very rapid (i.e., a few men reporting one week from diagnosis to curative surgery) compared with other cancers, perhaps facilitating the ability to minimize the emotional impact of the cancer experience.

In contrast, another participant reported that he identified strongly with Lance Armstrong, and was motivated by altruism to participate in our FTC study. A different subject stated that he and his wife work out frequently, that he is an adherent of physical fitness, stating, "I want to change my DNA." Another spoke of "the obligation of the cured," and of his plans to start a new support group to help others with TC.

#### Nature of emotional closeness

During CEGRM construction, most subjects had no difficulty in quickly and clearly identifying persons within their social network with whom they felt close. For the married men, this was most often their wives, whom they typically described as very close: "She means everything to me." Younger and unmarried men frequently cited "Mom" and friends. However, for some men there was a countervailing tendency to protect their mothers by keeping disturbing information from them, e.g., being secretive about details of the cancer diagnosis, treatment and sequelae. Brothers, especially those who had gone through similar TC experiences, were likely to be described as very close, e.g., "We brothers are tight. We all talk."

Many men had lifelong school friends who had become important confidantes over the years.

For many men, closeness meant doing pleasant and/or important activities together with one's friends, spouse or family, e.g., playing pick-up basketball, hunting, or working out at the gym, an action-oriented or emotive-avoidant coping style that many men adopted. Some men were information-oriented and sought out health information. Others used humor for coping, becoming the family clown to diffuse tensions, e.g., brothers often bantering with each other. Still others became "the strong, silent type," preferring to communicate their feelings, attitudes or preferences through actions rather than words.

Friendship bonds were important to the men in the study as indicated by the substantial number of colored symbols for social exchanges described above. Some participants designated groups rather than individual friends, e.g., "high school friends" or "Facebook friends." Others experienced friendship through recreational activities such as cook-outs, football, church picnics, hunting. One participant defined "friends" as "people outside the family to bounce things off". Another reported that his friends were helpful with negotiating the TC cancer experience, especially several who themselves were cancer survivors. One man summed it up: "buddies are supportive."

## Discussion

In general, we observed that the male FTC family members found the social assessment via the CEGRM process to be feasible, acceptable, and useful. Our participants revealed that they were more emotionally connected to their families-of-origin and current nuclear families than to extended family. We saw few communication blocks or family schisms between the men and their relatives. Most men participated in our clinical research out of altruism.

Initially, we were concerned that the CEGRM process might not be as acceptable or useful for men as it had been in women from HBOC families as reported in previous publications [25,33]. After completing the first few CEGRMs, we realized that the process was also working well with the men. Like the women that we had previously studied, the men were invariably cooperative, with mostly positive comments about the benefits of the CEGRM process, such as, "The CEGRM is very visual and physical; if you were just talking, you wouldn't have gotten all this," and "it [the CEGRM] helps to clarify relationships and priorities." Most men showed that they could quickly and easily grasp the concept and process of constructing the CEGRM, were comfortable with the interactive conjoint process, found it easy to talk about family and non-kin relationships in this active task-oriented context, and told a range of stories about selected aspects of their social relationships. However, the CEGRM was not for everyone, with one man confused about the purpose. Another participant, a traditional, elderly man, paradoxically stated, "I hate this sort of thing," after interacting in an animated way for an hour in the CEGRM process.

The construction time of the CEGRM depended on how expansive or guarded the men were in discussing their social milieu. The men varied widely in how much they perceived that they talked during the CEGRM process. Obviously, the man who reported talking 100% of the time was exaggerating on one level, but accurately reporting his subjective experience of being allowed to talk as much as he chose. The majority perceived that they talked more than half the time, which was a positive outcome of the process from our perspective of wanting to hear about their experiences in their own words and having the participants subjectively feel heard.

There was great intra- and inter-family variability in the parameters of interest, e.g., mood, communications, type and number of supports. Men asked to list friends, co-workers and others with whom they felt close often listed fewer non-kin than did women in HBOC families. Furthermore, the men might or might not give these friends the colored symbols denoting important social exchanges to the symbols designating their friends. This differed markedly from the women in HBOC and FTC families, for whom the decision to place the colored symbols was one of the criteria by which a friend was designated, e.g., "If I can't put a dot by these people, then they shouldn't be in my life."

### Emotional impact of FTC

Our finding that most TC survivors from our multiple-case families were well-adjusted, with only a few reporting distressing emotional issues, is in keeping with the general cancer literature. In one study, from 9% to 27% of TC survivors presented with anxiety or depression [34]. In a meta-analysis of mood disorder prevalence in cancer settings, there was less interviewer-defined depression and anxiety than anticipated in cancer patients, although a combination of various mood disorders occurred in 30-40% of hospitalized cancer patients [35]. In a study of long-term distress in a sub-set of men with *BRCA1/2 *mutations, predictors of distress included higher baseline distress and being unmarried [15], whereas, most of our distressed participants were married and all but one were affected with TC. The sources of the reported distress have yet to be resolved [6,36-38].

### Nature of emotional closeness and self-disclosure

Men's emotional supports play an important role in social and emotional adjustment to cancer risk and/or diagnosis. From our CEGRM data, it seemed that the men's emotional closeness with others was integrally entwined with tangible and informational supports as well as shared activities, i.e., instrumental support. Lay views of closeness generally consider it to depend on open sharing of information about oneself, i.e., self-disclosure. "The open sharing of the self (self-disclosure) does occur regularly among male friends, albeit to a varying degree." [39]. Specific elements of self-disclosure that may be important are the amount of personal information revealed, control over the conversational circumstances, topical breadth, and the emotional valence of the personal material disclosed. Since men often prefer topical discussions, it may take them longer to reach personally relevant information, e.g., we noted that many of our participants (who were either long-term survivors or their close relatives) considered themselves closest to long-term buddies from childhood, adolescence or young adulthood. Additionally, one participant remarked that it took a long time for him to make new friends after TC, given his workload, family obligations, etc. Indeed, while it seems by our observation that women with cancer reach self-disclosure sooner, men eventually do self-disclose emotion-laden information to those few people with whom they consider close. Perhaps researchers have not done enough longitudinal studies to capture men's longer time frame to reach this comfort zone.

### Men's Action- and task-orientation

One participant with prior TC stated his action-orientation this way: "We men are task-oriented. We act first, get diagnosis, second opinion, treatment, all while juggling work and family life obligations and maintaining normalcy of routine and everyday living. We perform the tasks necessary to self-preserve and outwardly present an image of a normal existence. Only after some time passes do we get around to processing the cancer; it takes a very long time to process verbally."

This definition of normalcy contrasts sharply with breast cancer patients, who report having many opportunities to process their cancer treatment decisions and report wanting to finish reconstructive surgery to feel normal again so that they can move on [40].

Some gender theories of masculinity suggest that being verbal about one's feelings is threatening to men, because the traditional masculine gender role is defined by being hard and strong [41-44]. However, during the FTC CEGRM process, the men appeared to talk freely, being very clear about their support system, freely and rapidly placing colored dots with apparently prompt and straightforward decision-making about who gets which specific colors. Thus, the interactive CEGRM process appears to be acceptable to and compatible with many men's public persona.

### Friendship bonds

Friendships play an important role in modern industrialized societies; friends, because of similarity in age, lifestyle and experience, are often useful at helping individuals adjust to many of life's challenges [45]. Friends, especially long-standing childhood or college friends, could provide valuable socio-emotional and tangible support, helping each other adjust during the rigorous challenges such as having cancer. However, spending time talking and in shared social activities may be inconsistent with the identity that men want to convey, the image of strong, masculine men who need minimal support [45]. This speculation has implications for future research and clinical care.

### Cancer survivor identity and normalcy issues

Cancer survivorship can be both an internal personal identity and also an external social identity. Men and women differ regarding how much they embrace these survivorship identities. Some men in our study warmly embraced the TC survivor identity, while others wanted nothing to do with being a cancer survivor, implying that self-identifying as a survivor was equated to being a victim and, therefore, being weak. There was a sense of wanting to restore one's dignity after the stressors of cancer treatment, and to connect with others like oneself to relieve the profound loneliness of being out-of-step with one's age-peers.

### Male gender identity and health

Despite the fact that more balanced gender role models are available for contemporary men, current research shows that men are still more likely than women to engage in dozens of health-related behaviors that increase the risk of disease, injury and death. Socialization continues to encourage risky behaviors for men and health promotion behaviors for women [46,47]. Group norms also guide behavior by providing information about normal behavior in social environments and constrain behaviors considered feminine, deviant, or off-limits. As a result, more traditionally masculine men who perceive barriers to healthy behaviors are less likely to report healthy behaviors [46]. As Courtney states in his editorial on men's health, men receive strong social prohibitions against doing anything that women do, and they are taught that health matters are women's concerns [41]. Thus, when a man brags that he hasn't been to a doctor in years, he is simultaneously describing a typically masculine "health practice" and also presenting himself as a real man [48].

We have evidence that risky behavior norms play out in FTC families. For example, we previously learned that men's adherence to doing testicular self examination (TSE) is suboptimal even within high-risk FTC families, and that its performance depends on physician recommendation, their relationship with the family physician, and testicular cancer worry [49,50].

### Study limitations and strengths

We recognize some limitations to our data interpretation in this first attempt to investigate social exchanges of men in FTC families. Our findings have limited generalizability since the study population was relatively homogeneous, namely, a white, heterosexual, educated, research-oriented group of families. Ascertainment was undoubtedly influenced by self-selection bias of participants willing to complete extensive epidemiological, medical, and psychosocial evaluations and travel to the NIH Clinical Center. We were unable to make any inferences about causality or directionality of observed associations, due to the cross-sectional design of our study. Data were available only from male participants; we had the opportunity to assess only 5 women and did not have the opportunity to interview most spouses, relatives, or friends. The sessions were not audio-taped, which might have yielded enhanced accuracy of quotations and other interaction dynamics. Both investigators who coconstructed CEGRMs with participants were female; we do not know if a male investigator would have uncovered more, less, or different material. Finally, we have attempted to accurately portray participants' views of their relationships with others, but did not have the opportunity to obtain the perspectives of most other people designated by our participants as exchanging social resources.

We believe that this pilot study has a number of strengths, and that it contributes significantly to the cancer genetics psychosocial literature. It was a sub-study of a larger multi-disciplinary team effort which benefitted from our varied clinical and research viewpoints as well as our collective synthesis of impressions. We focused on men and testicular cancer, two topics rarely addressed in cancer genetics psychosocial studies. Furthermore, we provide novel results of a systematic data collection method, applying a psychosocial assessment tool that plays to men's strengths. For these reasons, our pilot observations deserve follow-up.

### Clinical and counseling interventions

In light of findings from contemporary gender-study theories and findings of multiple factors affecting male health behaviors-such as seeking support and regular medical screening-it might be most effective for health education and genetic counseling interventions aimed at this population to be multi-modal. For example, educational programs might address health beliefs and knowledge; we are already providing this education about normal and abnormal testicular development and principles of inheritance within our FTC protocol [51]. Different methods such as peer groups, online support chats, or even the CEGRM process might provide feedback to participants about their male peers' health behaviors, e.g., that a lot of men perform regular TSE. There are currently many new online and social networking resources available for young adults with cancer:

• through a new National Cancer Institute (NCI) website for Young Adults

• (http://www.cancer.gov/cancertopics/aya/resources),

• the Testicular Cancer Resource Center (http://tcrc.acor.org)

• the LIVESTRONG Young Adult Alliance (http://www.livestrong.org/What-We-Do/Our-Actions/Programs-Partnerships/LIVESTRONG-Young-Adult-Alliance).

There are many ways of approaching strategies designed to facilitate coping with, and grieving over, testicular cancer [52]. Approaches likely to be most successful would incorporate and target male values, e.g., put emphasis on information-seeking and promoting active problem-solving. Since men tend to be "instrumental" rather than intuitively emotional grievers [53], providers offering follow-up services might ask "What did you do?" rather than "How did you feel?" (Perry Garfinkle, http://www.NYtimes.com/2011/7/26/health). It would be important to facilitate group belonging and to normalize health-promoting behaviors. On a practical level, men also value adding financial advice and advocacy into counseling agendas.

While recommendations regarding the efficacy of TSE are not uniform (due in large part to the remarkably high cure rates experienced even by men with widely-metastatic TC), there is, nonetheless, general agreement about the value of early detection through screening being preferable to later diagnosis, since the latter increases the likelihood that more toxic treatments (i.e., combination, platinum-based chemotherapy) will be required in addition to surgical treatment. Young men could be taught not only to do TSE and to seek medical attention when they notice a bodily change, and also encourage their brothers and friends to do the same. Furthermore, education could be combined with emphases on well-being, resilience and healthy development, positive psychology trends becoming more common in contemporary medicine and counseling.

Perhaps policy makers and public health officials also could address some of the social circumstances contributing to men's higher mortality and lower lifespan such as developing and promoting "men's health strategies" to balance out damaging media, institutional, and other forces that shape men's risk-inducing self-concepts and behaviors.

On the interpersonal level, marital relationships can become vulnerable in the face of intense stress of serious illness like TC [6,54]. Marriage and family therapy may be indicated for selected couples who have issues with body image, spousal emotional support and/or fertility concerns. However, we heard reports and saw the visual CEGRM evidence from our study participants that many FTC couples experienced supportiveness and closeness with their spouses as they worked through their TC experience. Feelings of heightened intimacy and bonding, satisfying sexual relations, and intimate involvement in the illness experience can contribute to overall adjustment [6,54]. Single men, a vulnerable group, will also undoubtedly also benefit from the opportunity to process their experiences over time [15]. Friendship relationships both in terms of long-term buddies from childhood and adolescence and those developed through members of a sports team, a comparatively neglected area of inquiry, could be further investigated as sources of social support, opinion molding and decision-making in familial cancer families as well as other male-associated cancers.

## Conclusion

We found that the CEGRM experience brought to the foreground men's emotional and social bonds which were not obvious in the existing cancer genetics psychosocial literature. This novel process of clients' talking while placing colored symbols on significant relationships, and emphasizing action over verbal reporting, uncovered previously under-appreciated material about social exchanges and inter-personal adjustment processes of men in FTC families. Participating men from FTC families reported that emotional closeness takes a long time to develop, but that long-standing relationships are an intrinsic part of their informational, tangible, and emotional supports. Although there are many gaps in our current knowledge about FTC, this is the first report opening up several areas of important inquiry in genetic counseling in FTC. Future research directions include advocating for longer follow-up of men with testicular cancer, using a variety of methodological modalities for psychological and social assessments, and performing social assessment CEGRMs in other cancer genetics study populations, such as men and women in families with Li-Fraumeni syndrome.

## Abbreviations

CEGRM: Colored Eco-Genetic Relationship Map; *CIS: Carcinoma in situ; *FTC: Familial Testicular Cancer; GU: Genito-Urinary; GWAS: Genome Wide Association Study; HBOC: Hereditary Breast-Ovarian Cancer Susceptibility Syndrome; NIH: National Institutes of Health, US; QoL: Quality of Life; HRQOL: Health Related Quality of Life; TM: Testicular Microlithiasis; TC: Testicular Cancer.

## Competing interests

The authors declare that they have no competing interests.

## Authors' contributions

JP was involved in every phase of the study from conceptualization and design to data collection, qualitative and quantitative analyses, writing, editing the manuscript. RK participated in conceptualization, design, qualitative analyses, writing, and editing. LH was involved primarily in data collection and editing. GG and CK contributed to data collection and editing. MHG contributed to conceptualization, design, resource management, and editing. All authors read and approved the final manuscript.

## Supplementary Material

Additional file 1 **Familial Testicular Cancer Epidemiology and Genetics Addenda **[55-76]. Click here for file
