# Supplementary material for: Close ties: an exploratory Colored Eco-Genetic Relationship Map (CEGRM) study of social connections of men in Familial Testicular Cancer (FTC) families
Source: Hered Cancer Clin Pract. 2012 Mar 1;10(1):2. doi: 10.1186/1897-4287-10-2 (PMC3323467; doi:10.1186/1897-4287-10-2)
Supplement: Additional file 1 — Familial Testicular Cancer Epidemiology and Genetics Addenda [55-76]. [file 1897-4287-10-2-S1.DOC]

**Appendix A- Familial Testicular Cancer Epidemiology and Genetics Addenda**

There are almost certainly multiple shared genetic and environmental causes of FTC [1], although a few environmental and occupational factors have been definitively implicated; *e.g.,* Swedish paper-mill workers and New Zealand firefighters [2-5]. Several possible biomarkers for subsequent development of TC are testicular microlithiases or trans-generational inheritance of an epigenetic event, e.g., differential LINE-1 methylation, that may be associated with disease risk and provide a mechanism by which environmental factors may influence clinical outcomes [6-7].

Testicular microlithiasis (TM) are testicular calcium deposits seen on ultrasound and appear to cluster in certain families, indicating a familial predisposition to TM itself as well as a possible link directly with TC [7-11]. TM has also been associated with various GU abnormalities [9].

The age at diagnosis for familial TM cases is, on average, 2-3 years younger than for the population cases in North America, United Kingdom, Australia and New Zealand; this younger age-at-diagnosis might be suggestive of a genetic basis for familial TC [12]. Familial patterns have been described that are compatible with autosomal dominant, recessive, X-linked recessive and multi-factorial inheritance. Hereditary syndromes or chromosomal anomalies, e.g., Klinefelter and Down syndromes, are known but rare causes of TC; the majority of familial cases being due to other causes. Searches for a single gene of high penetrance causing the majority of FTC have frustrated researchers using linkage studies for well over a decade, although some key loci-of-interest were discovered [13-15]. These preliminary findings led to further studies implicating multiple loci identified by GWAS [15-21]. Candidate TC susceptibility genes implicated in these studies include *KITLG, SPRY4, BAK1,* *TERT, DMRT1, and ATF71P,* as well as the *Y gr/gr deletion*. Kratz and colleagues took this a step further in proposing a method for a stratified genetic risk assessment for TC combining clinical risk factor identification with risk allele genotyping to form a combined clinical/genetic risk estimate for high risk screening [19].

Unlike Lynch syndrome or Hereditary Breast Ovarian Cancer (HBOC) syndromes in which deleterious gene mutations cause increased risk for a variety of malignancies, FTC seems largely organ-specific, affecting mainly testes and genito-urinary (GU) system development, although there is some evidence that incidence of germ cell ovarian tumors may also be modestly increased [22]. There is also recent evidence that differential LINE-methylation may be involved, providing a mechanism by which environmental factors may influence clinical

outcomes [6].

TC has become a paradigm of solid tumors that respond well to chemotherapy, with excellent survival rates of 95% five-year survival for early stage diagnosis [23] and 73% when there is distant metastasis at diagnosis [24]. Regrettably, our therapeutic success is offset by the emergence of significant long-term morbidity, including second malignant neoplasms, cardiovascular disease, neurotoxicity, nephrotoxicity, pulmonary toxicity, hypogonadism, decreased fertility, fatigue, and psychosocial problems, in a subset of treated patients. It is unclear whether there is a genetic basis for increased sensitivity to specific chemotherapy agents.

**References**

1. Heimdal K, Olsson H, Tretli S, Flodgren P, Borresen AL, Fossa SD: **Risk of cancer in relatives of testicular cancer patients**. *British Journal of Cancer* 1996, **73**(7):970-973.

2. Andersson E, Nilsson R, Toren K: **Testicular cancer among Swedish pulp and paper workers**. *American Journal of Industrial Medicine* 2003, **43**(6):642-646.

3. Bates MN, Fawcett J, Garrett N, Arnold R, Pearce N, Woodward A: **Is testicular cancer an occupational disease of fire fighters?** *American Journal of Industrial Medicine* 2001, **40**(3):263-270.

4. Aschim EL, Grotmol T, Tretli S, Haugen TB: **Is there an association between maternal weight and the risk of testicular cancer? An epidemiologic study of Norwegian data with emphasis on World War II**. *International Journal of Cancer* 2005, **116**(2):327-330.

5. Moline JM, Golden AL, Bar-Chama N, Smith E, Rauch ME, Chapin RE, Perreault SD, Schrader SM, Suk WA, Landrigan PJ: **Exposure to hazardous substances and male reproductive health: A research framework**. *Environmental Health Perspectives* 2000, **108**(9):803-813.

6. Mirabello L, Savage SA, Korde L, Gadalla SM, Greene MH: **LINE-1 methylation is inherited in familial testicular cancer kindreds**. *BMC Med Genet* 2010, **11**:77.

7. Korde LA, Premkumar A, Mueller C, Rosenberg P, Soho C, Bratslavsky G, Greene MH: **Increased prevalence of testicular microlithiasis in men with familial testicular cancer and their relatives**. *Br J Cancer* 2008, **99**(10):1748-1753.

8. Bates MN, Lane L: **Testicular Cancer in Fire Fighters - A Cluster Investigation**. *New Zealand Medical Journal* 1995, **108**(1006):334-337.

9. de Gouveia Brazao CA, Pierik FH, Oosterhuis JW, Dohle GR, Looijenga LH, Weber RF: **Bilateral testicular microlithiasis predicts the presence of the precursor of testicular germ cell tumors in subfertile men**. *J Urol* 2004, **171**(1):158-160.

10. Coffey J, Huddart RA, Elliott F, Sohaib SA, Parker E, Dudakia D, Pugh JL, Easton DF, Bishop DT, Stratton MR *et al*: **Testicular microlithiasis as a familial risk factor for testicular germ cell tumour**. *Br J Cancer* 2007, **97**(12):1701-1706.

11. Ringdahl E, Claybrook K, Teague JL, Northrup M: **Testicular microlithiasis and its relation to testicular cancer on ultrasound findings of symptomatic men**. *Journal of Urology* 2004, **172**(5):1904-1906.

12. Mai PL, Friedlander M, Tucker K, Phillips KA, Hogg D, Jewett MA, Lohynska R, Daugaard G, Richard S, Bonaiti-Pellie C *et al*: **The International Testicular Cancer Linkage Consortium: A clinicopathologic descriptive analysis of 461 familial malignant testicular germ cell tumor kindred**. *Urol Oncol* 2009.

13. Crockford GP, Linger R, Hockley S, Dudakia D, Johnson L, Huddart R, Tucker K, Friedlander M, Phillips KA, Hogg D *et al*: **Genome-wide linkage screen for testicular germ cell tumour susceptibility loci**. *Hum Mol Genet* 2006, **15**(3):443-451.

14. Rapley EA, Hockley S, Warren W, Johnson L, Huddart R, Crockford G, Forman D, Leahy MG, Oliver DT, Tucker K *et al*: **Somatic mutations of KIT in familial testicular germ cell tumours**. *Br J Cancer* 2004, **90**(12):2397-2401.

15. Rapley EA, Turnbull C, Al Olama AA, Dermitzakis ET, Linger R, Huddart RA, Renwick A, Hughes D, Hines S, Seal S *et al*: **A genome-wide association study of testicular germ cell tumor**. *Nat Genet* 2009, **41**(7):807-810.

16. Greene MH, Kratz CP, Mai PL, Mueller C, Peters JA, Bratslavsky G, Ling A, Choyke PM, Premkumar A, Bracci J *et al*: **Familial testicular germ cell tumors in adults: 2010 summary of genetic risk factors and clinical phenotype**. *Endocrine-Related Cancer* 2010, **17**(2):R109-R121.

17. Turnbull C, Rapley EA, Seal S, Pernet D, Renwick A, Hughes D, Ricketts M, Linger R, Nsengimana J, Deloukas P *et al*: **Variants near DMRT1, TERT and ATF7IP are associated with testicular germ cell cancer**. *Nat Genet* 2010, **42**(7):604-607.

18. Kanetsky PA, Mitra N, Vardhanabhuti S, Li MY, Vaughn DJ, Letrero R, Ciosek SL, Doody DR, Smith LM, Weaver J *et al*: **Common variation in KITLG and at 5q31.3 predisposes to testicular germ cell cancer**. *Nature Genetics* 2009, **41**(7):811-U865.

19. Kratz CP, Han SS, Rosenberg PS, Berndt SI, Burdett L, Yeager M, Korde LA, Mai PL, Pfeiffer R, Greene MH: **Variants in or near KITLG, BAK1, DMRT1, and TERT-CLPTM1L predispose to familial testicular germ cell tumour**. *Journal of Medical Genetics* 2011, **48**(7):473-476.

20. Kratz CP, Mai PL, Greene MH: **Familial testicular germ cell tumours**. *Best Practice and Research: Clinical Endocrinology and Metabolism* 2010, **24**(3):503-513.

21. Nathanson KL, Kanetsky PA, Hawes R, Vaughn DJ, Letrero R, Tucker K, Friedlander M, Phillips KA, Hogg D, Jewett MA *et al*: **The Y deletion gr/gr and susceptibility to testicular germ cell tumor**. *Am J Hum Genet* 2005, **77**(6):1034-1043.

22. Giambartolomei C, Mueller CM, Greene MH, Korde LA: **A mini-review of familial ovarian germ cell tumors: an additional manifestation of the familial testicular germ cell tumor syndrome**. *Cancer Epidemiol* 2009, **33**(1):31-36.

23. di Pietro A, de Vries EGE, Gietema JA, Spierings DCJ, de Jong S: **Testicular germ cell tumours: The paradigm of chemo-sensitive solid tumours**. *International Journal of Biochemistry & Cell Biology* 2005, **37**(12):2437-2456.

24. Bleyer A: **Latest Estimates of Survival Rates of the 24 Most Common Cancers in Adolescent and Young Adult Americans**. *Journal of Adolescent and Young Adult Oncology* 2011, **1**(1):37-42.
